# Single Cell Analysis of Neutrophils NETs by Microscopic LSPR Imaging System

**DOI:** 10.3390/mi11010052

**Published:** 2019-12-31

**Authors:** Riyaz Ahmad Mohamed Ali, Daiki Mita, Wilfred Espulgar, Masato Saito, Masayuki Nishide, Hyota Takamatsu, Hiroyuki Yoshikawa, Eiichi Tamiya

**Affiliations:** 1 Department of Applied Physics, Graduate School of Engineering, Osaka University, 2-1 Yamadaoka, Suita 565-0871, Japan; riyaz@uthm.edu.my (R.A.M.A.); daikimita0208@gmail.com (D.M.); wilfred@ap.eng.osaka-u.ac.jp (W.E.); yosikawa@ap.eng.osaka-u.ac.jp (H.Y.); tamiya@ap.eng.osaka-u.ac.jp (E.T.); 2 Department of Electric and Electronic Engineering, Universiti Tun Hussein Onn Malaysia, Parit Raja, Batu Pahat 86400, Johor, Malaysia; 3 Advanced Photonics and Biosensing Open Innovation Laboratory, AIST–Osaka University, Photonic Center Osaka University, Suita, Osaka 565-0871, Japan; 4 Department of Respiratory Medicine and Clinical Immunology, Graduate School of Medicine, Osaka University, 2-2 Yamadaoka, Suita, Osaka 565-0871, Japan; nishide@imed3.med.osaka-u.ac.jp (M.N.); thyota@imed3.med.osaka-u.ac.jp (H.T.)

**Keywords:** neutrophil, localized surface plasmon resonance (LSPR), microwell

## Abstract

A simple microengraving cell monitoring method for neutrophil extracellular traps (NETs) released from single neutrophils has been realized using a polydimethylsiloxane (PDMS) microwell array (MWA) sheet on a plasmon chip platform. An imbalance between NETs formation and the succeeding degradation (NETosis) are considered associated with autoimmune disease and its pathogenesis. Thus, an alternative platform that can conduct monitoring of this activity on single cell level at minimum cost but with great sensitivity is greatly desired. The developed MWA plasmon chips allow single cell isolation of neutrophils from 150 µL suspension (6.0 × 10^5^ cells/mL) with an efficiency of 36.3%; 105 microwells with single cell condition. To demonstrate the utility of the chip, trapped cells were incubated between 2 to 4 h after introducing with 100 nM phorbol 12-myristate 13-acetate (PMA) before measurement. Under observation using a hyperspectral imaging system that allows high-throughput screening, the neutrophils stimulated by PMA solution show a significant release of fibrils and NETs after 4 h, with observed maximum areas between 314–758 µm^2^. An average absorption peak wavelength shows a redshift of Δλ = 1.5 nm as neutrophils release NETs.

## 1. Introduction

Neutrophils have been considered for a long time as the principal soldiers of the innate immune system against invading pathogens. As primary immune cells to migrate to a site of inflammation, several defense mechanisms can be enacted to combat the spread of the disease such as phagocytosis of pathogens, degranulation, cytokine production, and formation of neutrophil extracellular traps (NETs) [1,2,3]. A report in 2004 by Brinkmann et al. [4], the mesh-like structure of NETs is found to be composed of histones and highly decondensed chromatin fibers [2] with varying diameters of 15 nm to 17 nm. Researcher Takei et al. [5] in 1996 has discovered that a pathway of cellular death that is different from apoptosis and necrosis. Though the framework of activation pathway is still under investigation, NETs are known to be released by activated neutrophils that form a fibrous network assembled from nuclear and granular components with larger size range [5]. This leads to the great potential of NETs as physical and antimicrobial barriers that first extracellularly restricts and then kills the pathogens at the site of inflammation. They can stay in the bloodstream for 6–8 h and in tissues for seven days [1]. However, this unique composition and characteristics of NETs are prone to be considered by our body as a threat. NETs are found in a variety of conditions aside from infection such as malignancy, atherosclerosis, and autoimmune diseases including rheumatoid arthritis (RA), systemic lupus erythematosus (SLE), anti-neutrophil cytoplasmic antibodies (ANCA)-associated vasculitis (AAV), psoriasis, and gout [6]. An imbalance between NET formation and the succeeding degradation are considered associated with autoimmune disease and its pathogenesis [6]. If left untreated or diagnosed late, prolonged exposure to NETs-related cascades associated to the autoimmunity could lead to systemic organ damage [6].

Evaluating if a patient has an autoimmune disease is conventionally performed with antinuclear antibody using immunofluorescence assay which requires at least two weeks to process due to the range of types associated to certain autoantibodies [7]. Aside from the long required time for this test and the cost; repeated dye staining is needed due to photobleaching effect and spectral overlap limited by available colors. A current emerging technology known as cytometry by time-of-flight (CyTOF) provides excellent cytoplasmic proteins tabulation in cell profiling as an alternative for this cell analysis purpose [8]. CyTOF method allows an extra stretch in a number of detection compared to fluorescence-based conventional flow cytometers; about 18 parameters of antigen. CyTOF uses transition elements isotopes as label surface markers with specific antibodies tag before introducing into the sample cells. Cells are further vaporized inside a coupled plasma (ICP) before the isotope-bound-cell entities are analyzed using time-of-flight mass spectroscopy. Even though this type of single cell analysis provides higher number of specific antibody detection, CyTOF method requires cells to be fixed before analysis, causing particular cells not available for continuous test acquisition [9]. Another technology that is directly targeted for NETs study is reactive oxygen species (ROS) measurement that is generated by nicotinamide adenine dinucleotide phosphate (NADPH) oxidase. Commonly used methods for this purpose are spectrophotometry and chemiluminescence [10]. However, their high activity, very short lifespan and extremely low concentration, makes ROS measurement a remaining challenge for researchers [11]. With this in mind, a localized surface plasmon resonance (LSPR) detection with integrated PDMS micro through-hole layer as microwells has been assembled that enables time-lapse single cell-level measurement and has the capability for continuous monitoring. 

Most conventional cell analyses methods involve bulk studies of cells that are considered having similar phenotypes. However, this type of study only provides the average values of the responses from highly heterogeneous populations of cells [12,13,14]. This approach can easily overlook unique cell responses which are important in leading to unique discoveries that could elucidate the cell behavior pathway [15]. Observation of any abnormal responses could provide new insights for early detection of infectious diseases, imbalanced protein secretion and for regular healthcare [16]. Therefore, it is highly crucial for researchers to focus on single cell analyses for studying intrinsic cellular responses.

LSPR-based biosensor has emerged as a promising technique for rapid detection of biomolecules, with great potential in diagnostic and point-of-care testing (POCT) applications [17,18,19,20]. This is made possible due to the interactions of biomolecules and sensing surface during the progression of LSPR. LSPR occurs when the frequency of the incident electromagnetic radiation matches the natural frequency of the electron cloud around the noble metal (e.g., gold, silver, copper) nanostructures, which leads to resonant oscillations of the electrons and sharp absorption of light at this frequency [21,22]. This optical property can also be affected by the shape and the size of the metal nanostructures which has been utilized in heavy metal detection [23,24,25]. Any variation in the refractive index (RI) near the nanoparticle surface due to biomolecules attachment, will lead to instantaneous changes in the LSPR-induced absorption peak wavelength. This could then provide high sensitive and real-time proteomic sensing capability [26,27]. As LSPR-based techniques may not require any multiple fluorescence staining procedures or secondary antibodies, they are highly favorable for real-time cell monitoring applications. Real-time monitoring of cells enables spontaneous cytoplasmic protein detection and monitoring at any given spatiotemporal domain. In addition, several studies have an emphasis on LSPR-based techniques for biomolecule detection through antigen-antibody interactions and protein surface binding kinetics [15,28,29,30,31]. More recently, LSPR-based observation of cellular activity aided by integration with microfluidics has also been reported which aid in doing high-throughput analyses [32,33,34].

In this work, fabricated chip is comprised of a perforated polydimethylsiloxane (PDMS) sheet that forms the microwell array (MWA) and a gold-capped nanopillar-structured cyclo-olefin polymer (COP) substrate that serves as the LSPR sensing platform. The MWA is prepared by thermal imprinting of an uncured PDMS between two silicon wafers with one surface having microposts pattern. This preparation method resulted in an easier fabrication process and a higher production repeatability compared to our previous study [35]. PDMS possesses a clear, non-toxic, and inert behavior on biological sample proving to be an excellent material to be used in various microfluidic applications. The use of the PDMS here allows forming inexpensive arrays of densely packed microwells to isolate individual cells into specific confinement. The microwell structures allow one to study any secreted proteomes released within the space without interfering with and to other cells using an optical observation instrument such as a hyper-spectral imaging system. The addition of hyper-spectral imaging platform provides a rapid, high-throughput, and continuous observation of heterogeneous responses of each individual cell. 

The LSPR substrate is also fabricated initially by thermal imprinting to produce the nanopillar structures on a COP film using a nanoporous anodic aluminum oxide (AAO) as mold. The substrate is then sputtered with gold (Au) to complete the LSPR substrate. The high-density arrays of nanoporous structure dimension can be formed by precisely controlling the anodizing parameters such as anodizing potential [35], substrate temperature, electrolyte solution [36] and pre-treatment of the alumina substrate. This has been used in LSPR-based sensing applications in our previous reports [35], including the multiplex screening of protein interactions using a hyperspectral imaging system [22]. Here, in order to assess the performance of the fabricated plasmon chips, their optical properties and the capacity to isolate single cells were investigated. Finally, the extent of fibril and NET release from neutrophils isolated on the chips were studied using the aforementioned hyperspectral imaging system.

In the current study, neutrophils obtained from whole-blood samples from healthy human donors were trapped, isolated, and stimulated on a novel microwell array (MWA) plasmon sensing chip without using pre immobilization of covalent interaction for NETs capturing purpose. Trapped single neutrophils cell inside MWA undergoes activation with phorbol 12-myristate 13-acetate (PMA) [5] which is a protein kinase C (PKC) agonist to activate NADPH-oxidase and reactive oxygen species (ROS) production [37]. Depending on the nature of the stimulus NADPH-oxidase can stimulate both apoptosis and NETosis (NET activation and release) [37] as in Figure S1. However, how ROS contribute to NETosis remains uncertain. Upon activation, the neutrophils will flatten and attach to the contacting surface ground and will lose its lobular morphology. Neutrophil elastase (NE) translocates to the nucleus upon escaping from azurophilic granules. It partially degrades certain histones causing chromatin decondensation to occur. Though the role remains unclear, myeloperoxidase (MPO) also assist NE in driving chromatin decondensation. Finally, the cell membrane ruptures and expel its mass of chromatin forming the NETs into the surrounding. There are other NETosis pathways but this is the prevailing view [38]. More importantly, the released NETs shall change the RI value and a corresponding LSPR shift (red shift) shall be observed. 

The focus of this study is the detection of the release of NETs from an observed isolated population but this platform is expected to be applicable to NET degradation monitoring as well. With this capability, an alternative method for NET related autoimmune disease testing platform could be realized. This report covers the assembly process of the chip and the initial results that indicate that our plasmon chips could provide in situ proteomic analysis and straightforward option for continuous high-throughput single-cell monitoring, not only limited to neutrophils but possibly for other types of cells as well.

## 2. Materials and Methods 

### 2.1. Fabrication of the Gold Sputtered Plasmonic Substrate

Self-organized nanoporous AAO mould was prepared for nanopillar structure formation on COP substrate. In this study, the nanoporous AAO mould was fabricated using a two-step anodizing method [39], which has been detailed in our previous reports [22,40,41,42,43]. High purity aluminum substrate with 4 mm thickness was annealed, parallel grained, acetone sonicated and then strip etched using chromic acid (Wako, Tokyo, Japan) before used. The first anodizing step was performed in 0.3 M oxalic acid at 80 V and 0 °C for 1 h, before etching in an aqueous solution containing phosphoric acid (1.16%, w/v) and chromic acid (5%, w/v) for 30 min at 70 °C. The second anodizing procedure was conducted at 60 V for 50 s and then exposed to a post-etch procedure in 0.23 M phosphoric acid for 12.5 min at 40 °C to widen the nanoporous structures.

Next, the nanoporous AAO mould was used to emboss its reverse pattern on a COP film (Zf-14, Zeon Corp., Tokyo, Japan) using thermal nanoimprinting (X-300H, SCIVAX Corp., Kanagawa, Japan). A fresh COP film (7.5 cm × 2.5 cm) was placed on top of the prepared aluminum oxide mold before being sandwiched between two identical 4″ inch silicon wafers. This allows uniform pressure distribution on COP film during thermal imprint method. Later, a hydraulic pressure of 2 MPa was applied while the temperature was kept constant at 160 °C for 10 min under vacuum condition. The transformed pattern on the COP film surface was further deposited with a 68 nm layer of gold using a magnetron sputtering (ACS4000, ULVAC system, Kanagawa, Japan) for faster and homogenous coating. The substrate was subsequently cut into smaller pieces (1 × 1 cm) and kept in a dry box until further use.

### 2.2. Fabrication of PDMS Microwell Array (MWA) Sheet

**Figure 1:**
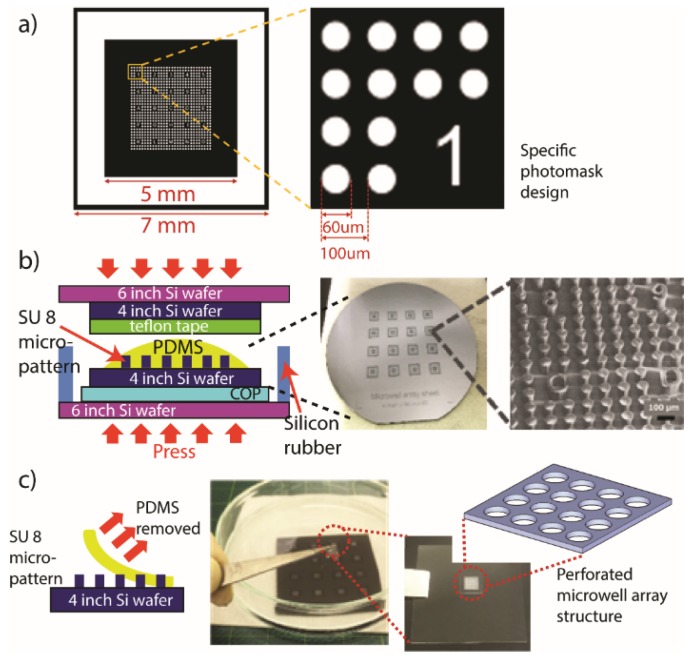
Fabrication of microwell array (MWA) sheet using thermal imprinting method. (**a**) Specific photomask design consists of microwell size of diameter 60 µm and pitch 100 µm was used to produce SU-8 design mold with a thickness of 60 µm height; (**b**) structure assembly before thermal imprinting procedure executed at 0.4 MPa of pressure with at 90 °C for 2 h. (**c**) Cured polydimethylsiloxane (PDMS) layer was carefully removed before replacing on the cover glass until further use.

Here, PDMS sheets with a varied thickness range of 30 µm, 60 µm, and 90 µm were prepared. Each sheet was designed to have perforated microwell array structures with diameter of 60 µm and 100 µm pitch as shown in Figure 1a. MWA sheets were prepared using a thermal imprinting procedure that utilizes a fabricated SU-8-patterned-based mold. To produce the mold, a specific photomask design was photolithographically transferred on a 60 µm-thick layer of SU-8 negative photoresist (SU-8 3050, MicroChem Corp., Westborough, MA, USA) coated on a silicon wafer. The unexposed region was removed using SU-8 developer (MicroChem Corp., Westborough, MA, USA) resulting to the structure in Figure 1a. 

The PDMS base agent (Silpot 184, Dow Corning Toray, Tokyo, Japan) was mixed with a curing agent in a 10:1 ratio before being degassed under vacuum. Uncured PDMS was coated onto the SU-8 mold, and then covered with a Teflon sheet (As One, Osaka, Japan) as shown in Figure 1b. About 0.4 MPa of pressure was applied at 90 °C for 2 h on this arrangement in thermal nanoimprinting (X-300H, SCIVAX Corp., Osaka, Japan). Then, the hardened and perforated PDMS sheet was carefully removed from the SU-8 mold as shown in Figure 1c and was temporarily placed on a cover glass (Matsunami Glass, Osaka, Japan) until further use.

### 2.3. Assembly of MWA Plasmon Chips

**Figure 2:**
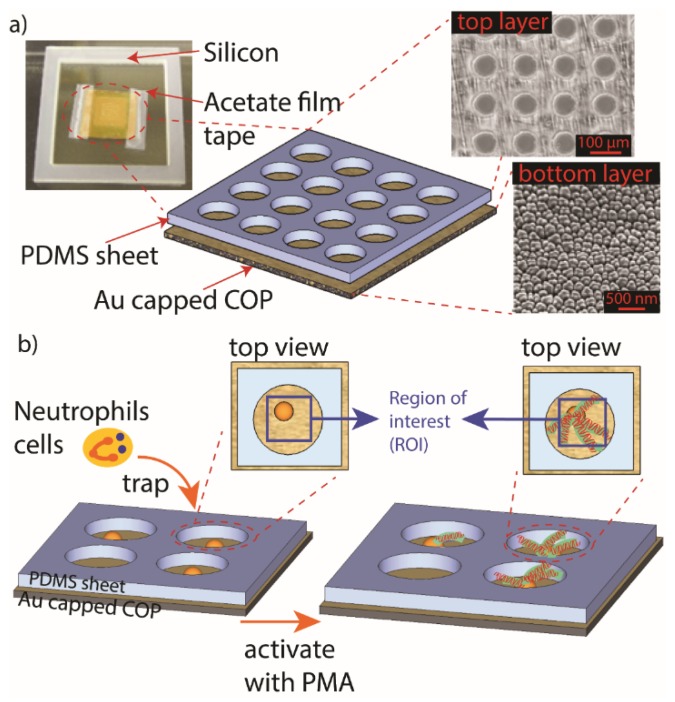
Full assembly of MWA plasmon chip. (**a**) (left) Perforated PDMS sheet with size of 7 mm × 7 mm was placed on 1 cm × 1 cm localized surface plasmon resonance (LSPR) sensing gold capped cyclo-olefin polymer (COP) substrate; (right) top view of MWA plasmon chip with well diameter of 60 µm. Bottom of the microwells has nanopillar structures. (**b**) Neutrophils cells are trapped into microwell array before stimulated with phorbol 12-myristate 13-acetate (PMA) solution. Released neutrophil extracellular traps (NETs) are observed using LSRP sensing chip.

Gold-sputtered plasmonic substrates were combined with PDMS MWA sheets in this study for isolating single cell and observing their release of fibrils and NETs. MWA sheets, as prepared in the previous section, were trimmed into 7 × 7 mm size pieces and then placed on top of gold sputtered plasmonic substrates as shown in Figure 2a. Silicone flow guards were placed around the assembled plasmonic chips to prevent any leakage of cell suspensions during observation.

### 2.4. Optimization of MWA Sheet Thickness

The as-prepared PDMS MWA sheets with thicknesses of 30 µm, 60 µm, and 90 µm were used to identify the optimum microwell height for single cell isolation by trapping fluorescent beads. About 150 µL of a solution containing fluorescent beads (ø = 15 µm, Thermo Fisher Scientific, Waltham, MA, USA) with excitation and emission wavelengths of 468 nm and 508 nm, respectively was dispersed in 0.1 % Tween 20 (Polyoxyethylene (20) Sorbitan Monolaurate, Wako, Japan) and was dropped on assembled MWA plasmon chips and allowed to sediment. After 30 min, excess beads were washed away and then the trapped beads were observed under a microscope to investigate the bead trapping capability and the presence of bubble formation with varying MWA sheet thickness.

### 2.5. Hyperspectral Imaging System

**Figure 3:**
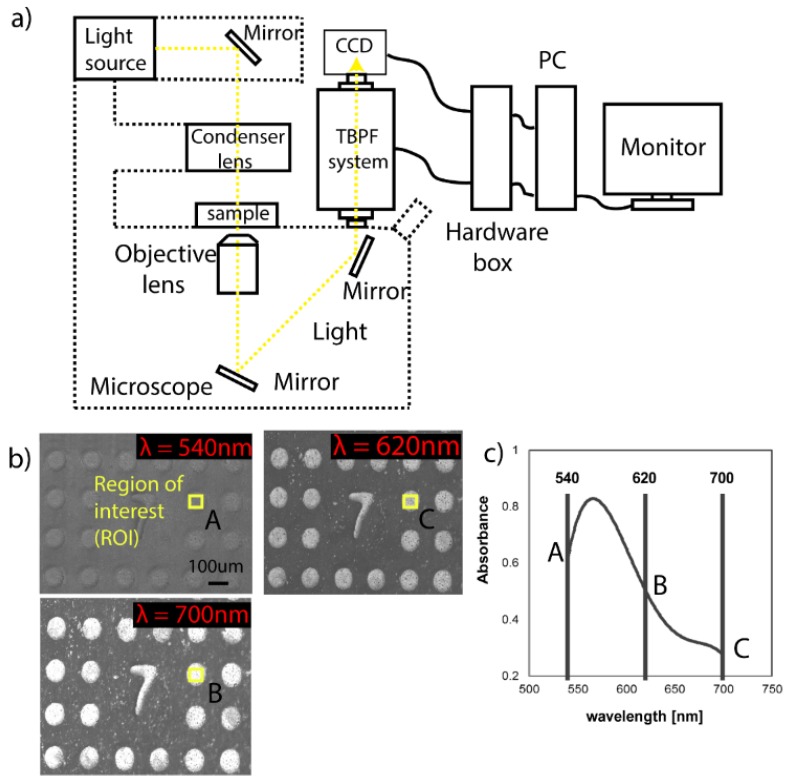
Hyperspectral imaging system setup: (**a**) Cross-section of the system; (**b**) substrate image corresponding to tunable bandpass filter (TBPF) system with wavelength from 540 nm to 700 nm with interval 0.5 nm. (**c**) Generated spectral graph response from 540 nm to 700 nm.

The configuration and operation procedure of our hyperspectral imaging observation system (Figure 3a) has been discussed in our previous work [22]. The optical imaging system in this study consists of a tunable bandpass filter (TBPF system), halogen light source (MegaLight 100, Mitutoyo, Kanagawa, Japan), air-cooled charged-coupled device (CCD) camera (BU-50LN, BITRAN Corp., Saitama-ken, Japan) and an inverted microscope (IX 81, Olympus, Tokyo, Japan). Incident light from the source passes through the substrate and to a 10× magnification lens (NA: 0.4, Olympus, Tokyo, Japan). Later, images from the plasmon chip are acquired and recorded by conducting on-frame image acquisition for each 0.5 nm interval between wavelengths of 540 to 700 nm, semi-automatically as shown in Figure 3b. Although the maximum imaging size capability is about 580 × 772 pixels, the region of interest (ROI) was fixed at 30 × 30 pixels. All imaging responses were managed using a custom-made LabVIEW (2014 SP1, National Instrument Corp., Austin, TX, USA) program as shown in Figure 3c. The entire wavelength measurement takes less than one minute to complete.

### 2.6. Extraction of Neutrophils from Raw Blood

About 5 mL of human blood was withdrawn from a healthy donor who gave his informed consent before the study was initiated. All procedures performed to harvest the cells are in compliance with the guidelines and regulations set by the Research Ethics Committee of Osaka University for the medical research targeting humans. Raw blood was collected in a sterilized vacutainer containing anti-coagulants and kept in an incubator for facility transfer purposes. Collected blood samples were processed within 15 min after collection.

Collected raw blood was transferred to a sterilized centrifuge tube before adding 5 mL of blood cell separation solution, Polymorphprep (Alere Technologies AS, Oslo, Norway). This tube was subjected to density gradient centrifugation at 400 G and 21 °C for 35 min (no brake was applied at the end). Four noticeable layers were formed: plasma, mononuclear cells (MC), polymorphonuclear leukocytes (PMN) and erythrocytes (in descending order). The PMN layer was then extracted to a new sterilized centrifuge tube. Dulbecco’s phosphate-buffered saline (D-PBS(–), Wako, Tokyo, Japan) was added to this tube until the final volume was 10 mL in total. A second centrifuge procedure was applied for 10 min for further PMN purification. The collected sediment pellet was then lysed using 1 mL distilled water by constant vortexing for exactly 30 s before diluting with D-PBS(–) to a total volume of 10 mL. This solution was then subjected to a third centrifugation and collection procedure. Finally, the cell suspension concentration was adjusted with D-PBS(–) prior to a full blood count procedure using a calibrated blood cell analyzer (XT-2000i, Sysmex, Hyogo, Japan). All usage of cell suspensions was restricted to within six hours of extraction for optimum observation results.

### 2.7. Neutrophils Isolation Using MWA Plasmon Chips

The neutrophil trapping and isolation capability of as-prepared MWA plasmon chips were evaluated by fluorescence imaging of cells trapped in the chips’ microwells for various cell suspension concentrations. Prepared cell suspensions were adjusted to concentrations of 2.0, 4.0, 6.0 and 8.0 × 105 cells/mL with D-PBS(–). Meanwhile, MWA plasmon chips were treated with oxygen plasma (PDC 21, Yamato Science, Tokyo, Japan) at 200 W for 10 s to induce hydrophilicity on the chip surface. About 150 µL of each cell suspension was dispensed on a series of plasmon chips and incubated for 30 min. Excess cells were washed with D-PBS(–) before introducing 4% paraformaldehyde (Wako, Tokyo, Japan) for 20 min to fix cells and disable any further release of biomolecules. About 5 µM of fluorescence staining agent (SYTOX Green, Thermo Fisher Scientific, Waltham, MA USA) was introduced for 5 min before washing with D-PBS(–). The isolation of neutrophils was evaluated based on high contrast imaging from a confocal laser microscopy system (A1Rsi, Nikon, Tokyo, Japan) and Plan Apo 10× objective lens (NA: 0.45, Nikon, Japan).

### 2.8. Verification of PMA Induced Neutrophils of Fibril Release

The as-prepared neutrophil suspensions (1.5 × 105 cells/mL) were used to study their release of fibrils under the presence of 100 nM PMA (Sigma Aldrich, St. Louis, MO, USA) for 2 h and 4 h. Extracellular structures were observed using fluorescence imaging at the end of each time period studied. Cells in D-PBS(–) with the same concentration served as the control. Each test suspension was dispensed separately at equal volume on a plane glass slide with surface treated with 0.001% poly-l-lysine (Wako, Japan). The surfaces were labelled as PMA(+)_2h, PMA(+)_4h, PMA(–)_2h and PMA(–)_4h. 

At the end of each time period, cell suspensions on the glass slide were fixed using 4% paraformaldehyde (Wako, Tokyo, Japan) for 15 min. The solution was washed off before staining the fixed cells with SYTOX green for 5 min. The stained cells were observed using the same confocal laser microscope system (A1Rsi, Nikon, Tokyo, Japan) with different setting of Plan Apo 60× objective lens (NA: 1.4 with oil immersion, Nikon, Tokyo, Japan). Based on the obtained images from each time period, the fibril sizes were analyzed. Each preparation and observation procedure was conducted with minimal disturbance to the cell suspensions.

### 2.9. Real-Time LSPR Observation of Neutrophil Extracellular Traps (NETs)

The as-prepared MWA plasmon chips were used to observe the real-time NET release of neutrophils using our hyperspectral imaging system. Suspensions (150 µL) containing about 6.0 × 105 cells/mL of neutrophils were dispensed on sterilized MWA plasmon chips. The LSPR responses from neutrophils in each chip were evaluated at 2 h and 4 h after exposing the cells to 100 nM PMA solution. Cells in DPBS(–) with the same concentration served as the control. The observed periods were labeled as PMA(+)_2H, PMA(+)_4H, PMA(–)_2H and PMA(–)_4H. The LSPR responses from each 30 individual neutrophils cells trapped in MWA plasmonic chip were recorded and analyzed. To confirm that the measured LSPR signal is indeed from the NETs released, the cells in the chips were fixed at the end of the measurement, stained with SYTOX green, and observed using the hyperspectral imaging system.

## 3. Results and Discussion

### 3.1. Optimization of MWA Plasmon Chips

**Figure 4:**
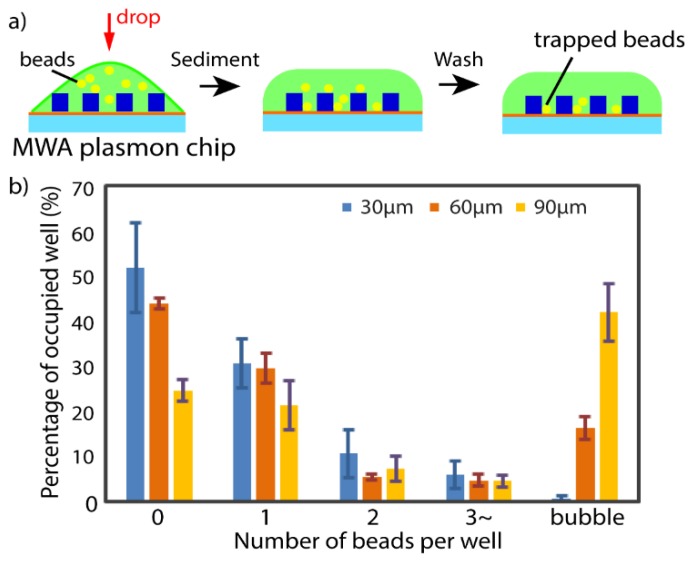
Optimization of 15 µm beads trapping over various thickness range: (**a**) About 150 µL of beads solution was dispersed on MWA plasmon chip before washing after 30 min; (**b**) beads trapping capability of varies thickness range of 30 µm, 60 µm, and 90 µm (error bar represents standard deviation with n = 3).

The PDMS sheet with microholes was successfully fabricated as shown in Figure 1b indicates that the setting used in the thermal imprinting procedures is at optimum. More importantly, the LSPR substrate with estimated width ranging between 140–150 nm and gap about 15 nm and having homogenous pillar dome shape (Figure 2) retained its characteristic LSPR spectra even with the addition of the PDMS sheet. The observed absorption peak of the LSPR substrate is at ≈560 nm (Figure 3b). The optimum thickness of the MWA sheet was determined based on the trapping capacity with 15 µm fluorescent beads (Figure 4a) in 0.1% Tween 20 solution. 

MWA sheets with several thicknesses (30, 60, 90 µm) were fabricated using the procedure described earlier. Figure 4b shows that the percentage of microwells containing zero beads decreases as the MWA sheet thickness increases (30 µm: 52%, 60 µm: 44%, 90 µm: 23%). Prior to washing, it was observed that all microwells contain microbeads. This suggests that, as the MWA sheet thickness increases, it is less likely for the trapped microbeads to be washed away. The washing of the beads outside the microwells don’t show dependence on the thickness of the MWA sheet. However, increasing the sheet thickness also led to an increase of bubble formation inside the microwells: 26% and 41% of microwells were found to contain bubbles for MWA sheets with thicknesses of 60 µm and 90 µm, respectively. On the other hand, the 30 µm sheet was found to contain no bubbles. This indicates that with an increase of height-to-diameter ratio, the air bubbles are easier to be trapped. This bubble formation problem was not observed when D-PBS(–) was used. This is related to the innate characteristic of a surfactant-containing solution that forms a thin plug deposit in a microchannel as a trailing film under low pressure [44]. As for the percentage of microwells containing single trapped bead, a decreasing trend with increasing sheet thickness (30 µm: 31%, 60 µm: 29% and 90 µm: 22%) was observed as shown in Figure 4b. Since the 30 µm sheet showed the highest percentage of wells containing zero trapped beads and the 90 µm sheet showed the highest percentage of wells containing air bubbles, the 60 µm MWA sheet was selected to be used in subsequent experiments. In addition, the percentage of microwells containing single trapped beads was almost the same for the 60 µm sheet as it was for the 30 µm sheet. Therefore, the optimized PDMS MWA thickness was determined to be at 60 µm.

### 3.2. Neutrophil Isolation Capability

**Figure 5:**
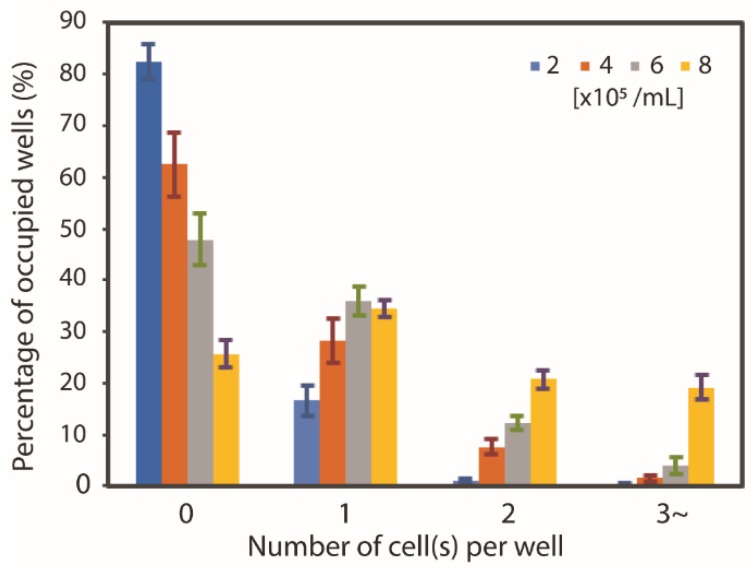
Neutrophils trapping efficiency using 60 µm thick MWA PDMS sheet (n = 3).

Suspensions of neutrophils at various concentration (2.0, 4.0, 6.0 and 8.0 × 105 cells/mL) were used to determine the optimum cell concentration for isolation of single cells in the as-prepared MWA plasmon chips. Figure 5 illustrates the result of the tests. The percentage of the microwells that were able to trap cells increased from 25.7% to 82.4% as the neutrophil cell concentration was increased. The increase in the number of cells per milliliter provides a higher possibility for cells to be isolated within the chips’ microwells. On the other hand, single cell isolation showed a gradual increase as the cell concentration increases before slightly dropping at the highest concentration of 8.0 × 105 cells/mL. Figure 5 also illustrates the single cell isolation percentage of each tested concentration: 2.0 × 105 cells/mL (16.6%), 4.0 × 105 cells/mL (28.5%), 6.0 × 105 cells/mL (36.3%) and 8.0 × 105 cells/mL (34.5%). From this study, it was found that the MWA plasmon chip shows the highest single cell isolation capability for the neutrophil suspension of 6.0 × 105 cells/mL with an average of 36.3% of wells being able to trap single cells (σ = 2.72%, N = 3). Therefore, the neutrophil suspension was determined to be the optimum at this concentration for use in subsequent experiments.

### 3.3. PMA Induced Fibril Release from Neutrophils

Neutrophils incubated with PMA solution undergo activation of nicotinamide adenine dinucleotide phosphate (NADPH) oxidase, an enzyme that produces reactive oxygen species (ROS), which in turn would result in activation of a protein—arginine deiminase 4 (PAD4) [37]. Activated PAD4 leads to chromatin decondensation with help from neutrophil elastase (NE) and myeloperoxidase (MPO) granules [38]. Chromatin is released into the cytosol and combines with cytosolic proteins. Within the first 4 h of stimulation, e.g., by PMA, it releases extracellular fibril or neutrophil extracellular traps (NETs), which can trap and disarm pathogens [4,38,45].

**Figure 6:**
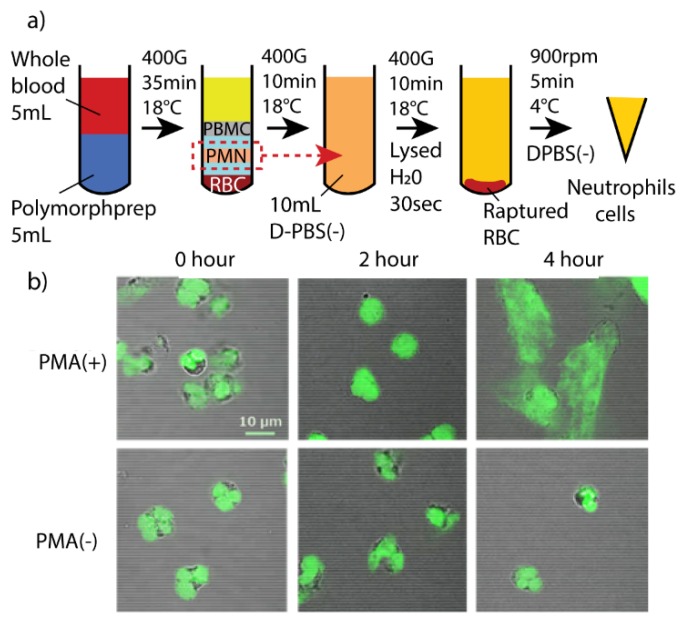
Phorbol 12-myristate 13-acetate (PMA) solution-induced neutrophils cells: (**a**) Preparation of neutrophils from human red blood cells; (**b**) the fibril releases from neutrophils cells under stimulation of PMA and D-PBS(–) solutions; denoted as PMA(+) and PMA(–), respectively.

Full blood counts revealed that over 90% of neutrophils were successfully collected using the previously mentioned protocol (Figure 6a). The collected neutrophils were then incubated with PMA solution to observe fibril release using fluorescence staining. Figure 6b shows the fluorescence images of neutrophils at 2 h and 4 h after incubation in PMA and in D-PBS(–) solutions; denoted as PMA(+) and PMA(–), respectively. The initial fluorescence image shows that on average, cells have areas no bigger than 101 µm^2^ (σ = 27.3 µm^2^, N = 30). The sizes remain almost the same for PMA(+)_2h, with a mean observed area of 113 µm^2^ (σ = 20.4 µm^2^, N = 30). However, neutrophils shows fibril formation after 4 h of incubation, with cells in PMA(+)_4h showing size between 314–758 µm^2^ (mean = 376 µm^2^, σ = 157 µm^2^, N = 30). On the other hand, the neutrophils’ sizes in PMA(–)_2h and PMA(–)_4h remain unchanged from their initial values. The results for PMA(+)_4h, as seen in Figure 6b, show similar structures and time-dependent characteristics that match with the above description.

### 3.4. Real-Time LSPR Imaging Observation of Neutrophils

**Figure 7:**
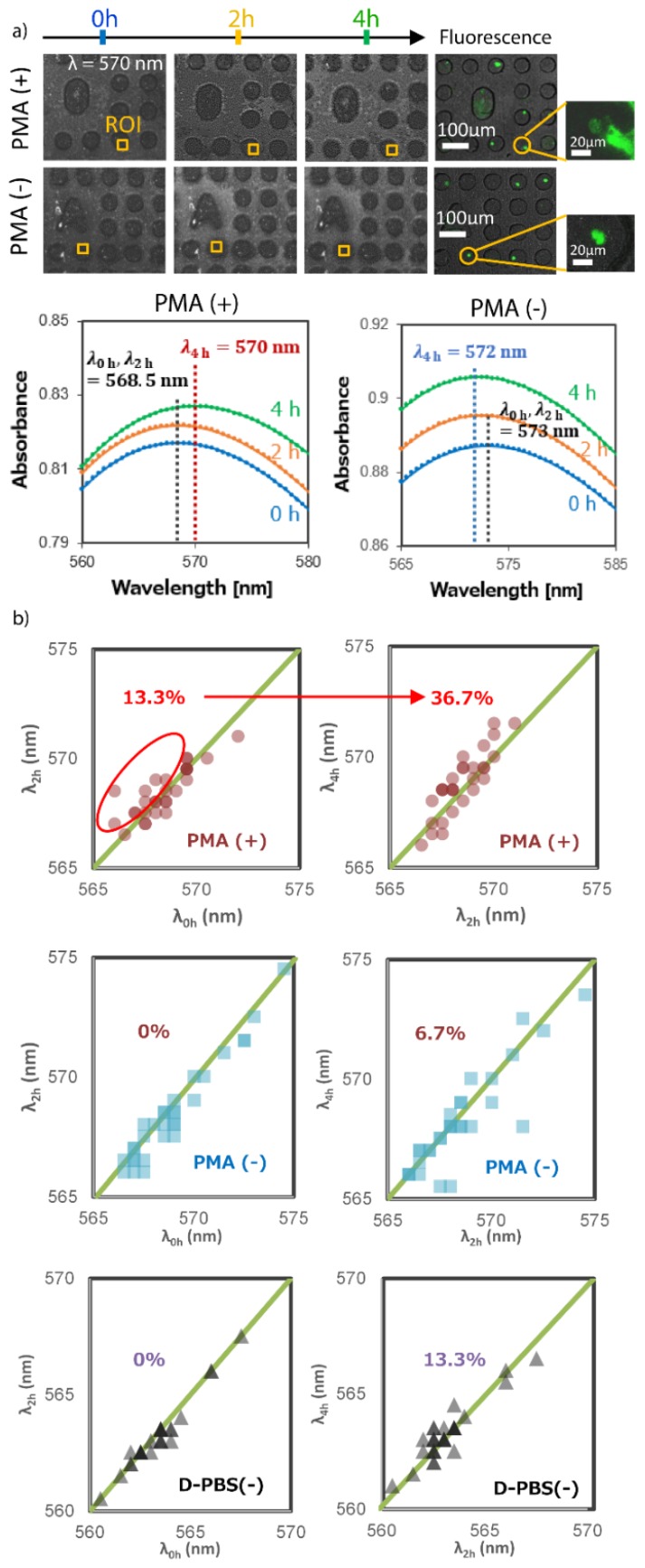
The real-time LSPR observation of single neutrophils cells: (**a**) The real-time LSPR observation of single neutrophils cells trapped in microwell at 570 nm wavelength over 4 h; (**b**) the distribution shift of 30 individual microwells with neutrophils and without neutrophils.

Suspensions containing neutrophils were introduced to MWA plasmon chips and stimulated by PMA solutions before observation under our hyperspectral imaging system. Neutrophils incubated in D-PBS(–) was used as the control in this study. A chronological observation was performed at 2 h and 4 h after incubation with PMA and D-PBS(–); denoted as PMA(+) and PMA(–), respectively. Figure 7a shows the acquired and processed spectroscopic images of the 30 × 30-pixel region of interest. The LSPR absorption peak shifts of 30 random cells were analyzed and also presented in Figure 7b. The color intensity of the points in the graph indicates the number of overlapping points.

The average absorption peak wavelengths for PMA stimulated neutrophils at each measurement time are PMA(+)_0h = 568.5 nm, PMA(+)_2h = 568.5 nm and PMA(+)_4h = 570.0 nm. A redshift of Δλ = 1.5 nm was observed after 4 h of incubating the neutrophils with PMA. This redshift was largely due to the increase in the neutrophils fibril release that cause changes in the refractive indices inside their respective microwells. As the fibrils cover the surface, an increase in refractive index is expected resulting to the observed redshift of absorption peak. For confirmation, the neutrophils stained by SYTOX green fluorescence image in PMA(+)_4h shows an expanded released NET that is consistent with Figure 6b. On the other hand, neutrophils incubated with D-PBS(–) showed an average absorption peak wavelengths of 573 nm, 573 nm and 572 nm for PMA(–)_0h, PMA(–) _2h and PMA(–)_4h, respectively. An average blue shift of Δλ = 1 nm was also observed.

A blue shift is expected when there as a decrease in refractive index on the observed surface. This observed phenomenon is associated to the shrinking of the cells as they undergo cell death and possible detachment from the contact surface. In addition, though not explored in this study, the blue shift of the absorption spectra can be monitored to determine the degradation of the NETs with a different stimulus. This can be proven useful in future NETosis studies for the elucidation of activation and degradation pathways.

Further analyses of 30 cell samples show similar absorption peak shifts, as summarized in the scattergram in Figure 7b. About 13.3% of neutrophils in PMA(+)_2h show red shift in peak absorbance. This figure increases to 36.7% for neutrophils in PMA(+)_4h. Meanwhile, the neutrophils in D-PBS(–) demonstrate no change after 2 h in the observed average absorption peak wavelength. There was a slight increase of about 6.7% for neutrophils with redshifted absorption peak wavelength after 4 h which indicates the release of NETs or the swelling of cells that increases the initially covered area. A different activation pathway of NETs must have been triggered which could be related to ROS production but this was not explored in the current study. More importantly, the majority of the cells in D-PBS(–) produced blue shifted absorption peaks which indicate that most cells didn’t release NETs and that a decrease in cell size or detachment from the contact surface occurred. These result clearly indicate that the MWA plasmonic chips used in this study can be utilized for real-time and label- free analyses of fibril and NET release from neutrophils and can clearly demarcate them from inactive neutrophils.

To demonstrate that the observed absorption peak shift is specific to the NETs released, a MWA plasmon chip was tested with D-PBS(–) only. It can be observed in Figure 7b that no significant shift of absorption peak spectra was observed after 2 h of exposure to D-PBS(–). However, redshifted absorption peaks were observed after 4 h. This is associated with the uncured PDMS silicone oil from the MWA sheets that seep out as the time progresses. This problem has already been minimized based on the several washing attempt. It is important to note that the absorption peak observed and the corresponding shifted peak fall below 565 nm which is lower than the observed peaks from the case of neutrophils. This indicates that the effect of the peak shift caused by the oil can be considered insignificant and that the observed peak shifts from NETs are specific.

It is estimated that at most 0.1–0.5% of neutrophils seems to have NET formation, so that 10–50 NET-formed neutrophils may be detected in a drop of blood (1 µL) from a healthy patient. In the NETosis-associated diseases, including infection, vasculitis, and auto-inflammatory diseases, the number of NET-formed neutrophils is predicted to increase (at least 0.5–2%; 50–200/µL). Linking the red blood cell lysis procedure, we believe that our LSPR-based analyzing system may allow to know the real-time status of NETosis of patients by 1 drop of blood sample in future.

Regarding clinical applications, the following clinical issues have not yet been elucidated. 1; depending on the disease, how much NET-forming neutrophils increase, 2; whether (pathogen-associated molecular patterns (PAMPs) or damage-associated molecular patterns (DAMPs) stimulation causes neutrophils from patients to respond more than neutrophils from healthy donors, and 3; whether neutrophil reactivity is impaired in neutrophils from cancer patients. We believe that analyzing the NET-forming potential in a single cell resolution using our LSPR-based assay system may enable to clarify these clinical questions in future.

Although the detection of NETs released was successfully demonstrated, the system still needs improvement for clinical applications. The current device has the potential to trap and prepare the desired number of cells but the LSPR measurement is limited by the image acquisition hardware and the analysis software. As larger area is measured, a longer acquisition time will be needed. A better hyperspectral imaging system is still desired and will be the target of improvement in the future. 

## 4. Conclusions

MWA sheets integrated with plasmonic sensing capability for studies of single neutrophils have been successfully investigated using our hyperspectral imaging system. The fabrication of the MWA layer using PDMS has allowed for trapping of single neutrophils before continuous analysis using LSPR. The thickness of the PDMS MWA sheet has been optimized with 60 µm thickness for greatest single cell isolation with minimal trapped bubbles. The optical image of the MWA sheets showed the successful fabrication of smooth perforated sheets based on the optimized procedure. The best single neutrophil isolation using the as-prepared MWA plasmonic chips was achieved for a concentration of 6.0 × 105 cells/mL, with 36.3% trapping capability of single cells; 105 microwells with single cell available for study. Investigations under a confocal microscope revealed that neutrophils stimulated by 100nM PMA solution show significant release of extracellular fibrils and NETs after 4 h with maximum observed areas between 314–758 µm^2^. Average LSPR absorption peak wavelengths showed a red shift of Δλ = 1.5 nm as neutrophils released NETs. This redshift was observed from 36.7% of imaged cells after 4 h of stimulation. In addition, the platform allows the identification of inactive neutrophils based on the blueshifted absorption peak. In addition, the degradation of the NETs could be studied which could further expand the application of this detection platform. With these, a label-free plasmon chip for high-throughput single cell detection of released NETs has been realized that could aid in studying NETosis for autoimmune disease detection and pathogenesis elucidation. This platform can also be proven useful for other studies with cell behavioral traits that can affect the optical density of the surrounding environment like stem cell differentiation, tumor cell release progression, co-culture screening, cell growth and death monitoring, and many other more.

## Supplementary Materials

The following are available online at https://www.mdpi.com/2072-666X/11/1/52/s1, Figure S1: The Neutrophil mechanism as activated with phorbol 12-myristate 13-acetate (PMA) solution.
