# Supplementary material for: Single Cell Analysis of Neutrophils NETs by Microscopic LSPR Imaging System
Source: Micromachines (Basel). 2019 Dec 31;11(1):52. doi: 10.3390/mi11010052 (PMC7019790; doi:10.3390/mi11010052)
Supplement: Supplementary file 1 [file micromachines-11-00052-s001.pdf]

micromachines

Supplementary Materials: Single Cell Analysis of Neutrophils by Microscopic LSPR Imaging System

Riyaz Ahmad Mohamed Ali, Daiki Mita, Wilfred Espulgar, Masato Saito *, Masayuki Nishide, Hyota Takamatsu, Hiroyuki Yoshikawa and Eiichi Tamiya

Neutrophil Extracellular Traps Release (NETs):

Neutrophils cells play an important role for a strong immune system. Neutrophils are able to migrate to the infection site before engulfing and killing any harmful pathogens. Neutrophils serve as host defense in human body by phagocytosis, degranulation, cytokine production and neutrophil extracellular traps (NETs) production. The neutrophil mechanism is shown in illustration Figure S1. The NETs formation starts with stimulated neutrophil cell that leads to activation of nicotinamide adenine dinucleotide phosphate (NADPH) oxidase complex and reactive oxygen species (ROS) production which results in activation of protein-arginine deiminase 4 (PAD4). With the help of neutrophil elastase (NE) and myeloperoxidase (MPO), chromatin decondensation occurs. This causes nuclear membrane to break and the chromatin be released outside of the cell through membrane pores and cellular lysis. This rapture is known as NETs formation.

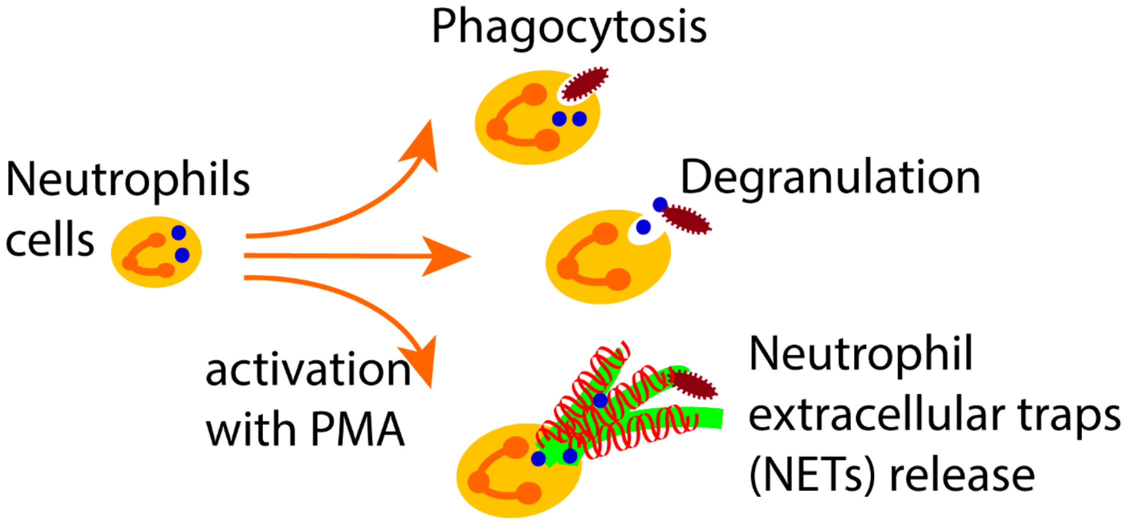

Figure S1. The Neutrophil mechanism as activated with phorbol 12-myristate 13-acetate (PMA) solution.

© 2019 by the authors. Licensee MDPI, Basel, Switzerland. This article is an open access article distributed under the terms and conditions of the Creative Commons Attribution (CC BY) license (http://creativecommons.org/licenses/by/4.0/).
